# The association between socioeconomic factors and weight loss 5 years after gastric bypass surgery

**DOI:** 10.1038/s41366-020-0637-0

**Published:** 2020-07-10

**Authors:** Erik Stenberg, Ingmar Näslund, Carina Persson, Eva Szabo, Magnus Sundbom, Johan Ottosson, Erik Näslund

**Affiliations:** 1 Department of Surgery, Faculty of Medicine and Health, Örebro University, Örebro, Sweden; 2 Department of Community Medicine and Public Health, Faculty of Medicine and Health, Örebro University, Örebro, Sweden; 3 Department for Sustainable Development, Region Örebro County, Örebro, Sweden; 4 Department of Surgical Sciences, Uppsala University, Uppsala, Sweden; 5 Division of Surgery, Department of Clinical Sciences, Danderyd Hospital, Karolinska Institutet, Stockholm, Sweden

**Keywords:** Obesity, Risk factors

## Abstract

**Introduction:**

Patients with low socioeconomic status have been reported to have poorer outcome than those with a high socioeconomic status after several types of surgery. The influence of socioeconomic factors on weight loss after bariatric surgery remains unclear. The aim of the present study was to evaluate the association between socioeconomic factors and postoperative weight loss.

**Materials and methods:**

This was a retrospective, nationwide cohort study with 5-year follow-up data for 13,275 patients operated with primary gastric bypass in Sweden between January 2007 and December 2012 (*n* = 13,275), linking data from the Scandinavian Obesity Surgery Registry, Statistics Sweden, the Swedish National Patient Register, and the Swedish Prescribed Drugs Register. The assessed socioeconomic variables were education, profession, disposable income, place of residence, marital status, financial aid and heritage. The main outcome was weight loss 5 years after surgery, measured as total weight loss (TWL). Linear regression models, adjusted for age, preoperative body mass index (BMI), sex and comorbid diseases were constructed.

**Results:**

The mean TWL 5 years after surgery was 28.3 ± 9.86%. In the adjusted model, first-generation immigrants (%TWL, B −2.4 [95% CI −2.9 to −1.9], *p* < 0.0001) lost significantly less weight than the mean, while residents in medium-sized (B 0.8 [95% CI 0.4–1.2], *p* = 0.0001) or small towns (B 0.8 [95% CI 0.4–1.2], *p* < 0.0001) lost significantly more weight.

**Conclusions:**

All socioeconomic groups experienced improvements in weight after bariatric surgery. However, as first-generation immigrants and patients residing in larger towns (>200,000 inhabitants) tend to have inferior weight loss compared to other groups, increased support in the pre- and postoperative setting for these two groups could be of value. The remaining socioeconomic factors appear to have a weaker association with postoperative weight loss.

## Introduction

Gastric bypass surgery is a safe and effective treatment for morbid obesity [1, 2]. Mean weight loss remains high even after long-term follow-up [3]. There are groups of patients, however, that experience a lesser degree of long-term weight loss [4]. While age, sex and obesity-related comorbidities, such as diabetes, have been reported to influence postoperative weight loss [5–10], the influence of socioeconomic factors remains unclear [11, 12]. A low socioeconomic status has been reported to be associated with higher complication rates and poorer outcomes after surgical procedures [13–15]. Recent studies have shown the same applies to gastric bypass surgery, with an increased risk for postoperative complications and less improvement in quality of life [16, 17]. The recognition of risk factors for inadequate postoperative weight loss that can be identified preoperatively may help in identifying certain groups of patients who require increased support in the pre- and postoperative setting.

The aim of the present study was to identify socioeconomic factors associated with suboptimal postoperative weight loss 5 years after surgery.

## Methods

The Scandinavian Obesity Surgery Register (SOReg) is a nationwide register for metabolic surgery, containing virtually all patients operated with metabolic surgery in Sweden since 2007 [18]. From the SOReg, all primary gastric bypass procedures from June 1, 2007 until December 31, 2012, were identified and assessed for inclusion in the study. Pre-established exclusion criteria were age <18 years; missing information on weight 5 years after surgery; and operation at a centre not routinely performing a 5-year follow-up. Based on personal identification numbers (unique to all Swedish citizens), data from SOReg were cross-linked to the Swedish National Patient Register, the Swedish Prescribed Drug Register, and Statistics Sweden. The Swedish National Patient Register covers inpatient and outpatient care with high validity for the variables included in the present study [19]. The Prescribed Drug Register covers all prescribed drugs in Sweden, based on ATC-codes [20].

Baseline characteristics, perioperative data, and follow-up data were obtained from the SOReg, the Swedish National Patient Register and the Swedish Prescribed Drug Register.

Patient-specific data on socioeconomic factors (education, profession, disposable income, residence, marital status, financial aid, and heritage) were obtained from Statistics Sweden, reporting quality assured and validated personal data on socioeconomic factors (https://www.scb.se/en/About-us/main-activity/quality-work/statistics-sweden-has-quality-certification/). Educational level was divided into four groups based on the highest completed education at the time of surgery: primary education (≤9 years of schooling), secondary education (completed 11–12 years of schooling), higher education ≤3 years (completed college or university degree with ≤3 years of education), and higher education >3 years. Profession was reported in accordance with the International Standard Classification of Occupations from 1988 (ISCO-88) and further classified into the following subgroups (based on the respective ISCO-88 groups): Senior officials and management (group 1), Professionals and technicians (groups 2 and 3), Clerical support workers (group 4), Service and sales workers (group 5), Manual labour (groups 6–8), and Elementary occupation (group 9). The place of residence was divided into three categories: Large city (>200,000 inhabitants) and municipality near a large city, medium-sized town (≥50,000 inhabitants) and municipality near a medium-sized town, and smaller town or urban area (<50,000 inhabitants) and rural municipality disposable income, in accordance with the definition of the Swedish Association of Local Authorities and Regions. Disposable income (total taxable income minus taxes and other negative transfers) was divided into percentiles (lowest 20th, 20th to median, median to 80th, and highest 80th) based on the disposable income of all adults in Sweden during the year of surgery. Marital status, financial aid, and heritage were all based on accepted standards as described previously [16].

Comorbidity at baseline was defined as continuous treatment (pharmacological or with positive airway pressure) for sleep apnoea, hypertension, dyslipidaemia, dyspepsia/GERD, and depression. Diabetes was defined according to the American Diabetes Association [21]. Cardiovascular comorbidity was defined as a diagnosis of ischaemic heart disease, angina pectoris, arrhythmia, or heart failure at any time prior to surgery.

### Procedure

The surgical technique for laparoscopic gastric bypass is highly standardized in Sweden, with the majority being antecolic, antegastric, Roux-en-Y gastric bypass with a small (<25 mL) gastric pouch, an alimentary limb of 100 cm and a biliopancreatic limb of 50 cm [22]. In open cases, the gastric pouch and small bowel are handled similarly.

### Outcome

The main outcome was weight loss 5 years after surgery defined as the percentage of total weight loss (%TWL). Secondary outcomes were percentage excess BMI loss (%EBMIL = 100 × [preoperative BMI – BMI 5 years after surgery]/[preoperative BMI – 25]), and the proportion of patients achieving satisfactory weight loss (defined as EBMIL ≥ 50%).

### Sensitivity analysis

Risk factors for loss to follow-up were analyzed as a sensitivity analysis. A further analysis was performed including only patients operated on at centres with >75% follow-up rates for the same year of surgery.

### Statistics

Categorical values were presented as numbers and percentages, continuous values as mean ± standard deviation for values with normal distribution, and median with interquartile range (IQR) for values without normal distribution. The association between patient-specific risk factors and weight loss was evaluated using linear regression analyses with the regression coefficient (*B*) and 95% confidence interval as measures of association. The socioeconomic factors were further evaluated in a linear regression model adjusted for preoperative factors (age, BMI, sex, and comorbidity) known to influence weight loss.

The association between patient-specific risk factors and the chance of achieving an EBMIL ≥ 50% was evaluated with logistic regression. All factors evaluated were also entered into a multivariable logistic regression model. The model was also tested for multicollinearity using linear regression. A variance inflation factor (VIF) >5 was considered to indicate an issue with multicollinearity.

Due to the multiplicity of variables analyzed, the Bonferroni–Holm method was used to compensate for multiple calculations [23].

IBM SPSS version 25 (IBM Corporation, Armonk, New York, USA) was used for all statistical analyses.

## Results

During the inclusion period, 29,524 patients operated with a primary gastric bypass procedure were identified. After exclusion of patients who died before the 5-year follow-up (*n* = 336), patients operated on at a centre not routinely performing a 5-year follow-up (*n* = 4326), and patients without weight registered at the 5-year follow-up (*n* = 11,587), 13,275 patients remained within the study group (53.4% of patients with potential 5-year follow-up).

### Operative data and weight results

The mean age at surgery was 42.3 ± 11.1 years, the mean preoperative BMI was 42.5 ± 5.3 kg/m^2^, 77.6% were women and 49.8% suffered an obesity-related comorbid condition.

In all, 94.6% of the operations were managed with a laparoscopic approach (*n* = 12,561), 1.3% were converted to open surgery (*n* = 167), and 4.1% were primarily open procedures (n = 547). The mean operation time was 84 ± 38.9 min, with a median postoperative hospital stay of 2 days (IQR 2–3 days).

At 1, 2, and 5 years after surgery, the mean BMI was reduced to 29.2 ± 4.6 kg/m^2^, 28.8 ± 4.8 kg/m^2^, and 30.4 ± 5.3 kg/m^2^, respectively (*p* < 0.0001 for all, compared to baseline). At 5 years, the average reduction in BMI was 12.1 ± 4.8 BMI units, corresponding to an average percentage %TWL of 28.3 ± 9.9%, and a %EBMIL of 71.6 ± 26.1%. At that time point, satisfactory excess weight loss (≥50% EBMIL) was achieved in 10,572 patients (79.6%).

### Factors affecting postoperative weight loss at 5 years

Lower %TWL was associated with an occupation other than service and sales work, higher disposable income, living in larger cities, receiving financial aid other than social benefits, and being a first-generation immigrant, as well as older age, male gender, and obesity-related comorbidities. Higher %TWL was seen in higher BMI and single status (Table 1).

**Table 1:** Percentage total weight loss 5 years after surgery.

	*N*	%TWL	*B* (95% CI)	Unadjusted *p*
Age				
<30	1877	31.1 ± 10.59	Reference	Reference
30–40	3474	29.7 ± 9.79	−1.4 (−2.0 to −0.9)	<0.0001*
40–50	4303	27.8 ± 9.57	−3.3 (3.8 to −2.8)	<0.0001*
50–60	2798	26.5 ± 9.22	−4.7 (−5.2 to −4.1)	<0.0001*
>60	823	24.9 ± 9.43	−6.2 (−7.1 to −5.4)	<0.0001*
BMI				
<40	4617	26.5 ± 9.17	Reference	Reference
40–50	7521	29.0 ± 9.88	2.5 (2.2–2.9)	<0.0001*
50–60	1048	30.6 ± 10.90	4.1 (3.4–4.7)	<0.0001*
>60	89	32.1 ± 13.71	5.5 (3.6–7.5)	<0.0001*
Sex				
Female	10,308	28.9 ± 9.83	Reference	Reference
Male	2967	26.1 ± 9.63	−2.8 (−3.2 to −2.4)	<0.0001*
Comorbidity				
Sleep apnoea	1275	26.4 ± 9.91	−2.1 (−2.7 to −1.6)	<0.0001*
Hypertension	3574	26.3 ± 9.52	−2.7 (−3.1 to −2.3)	<0.0001*
Diabetes	2604	25.1 ± 9.50	−3.9 (−4.4 to −3.5)	<0.0001*
Dyslipidaemia	1412	25.6 ± 9.73	−3.0 (−3.5 to −2.5)	<0.0001*
Dyspepsia/GERD	1053	27.5 ± 9.96	−0.9 (−1.5 to −0.3)	0.006
Depression	1731	26.9 ± 11.04	−1.6 (−2.1 to −1.1)	<0.0001*
Cardiovascular comorbidity	705	26.2 ± 9.97	−2.2 (−3.0 to −1.5)	<0.0001*
Education				
Primary education < 9 years	2232	28.4 ± 10.31	−0.1 (−0.5 to 0.4)	0.720
Secondary education	8154	28.5 ± 9.78	Reference	Reference
Higher education < 3 years	1408	27.8 ± 9.73	−0.7 (−1.3 to −0.2)	0.012
Higher education > 3 years	1425	27.9 ± 9.54	−0.5 (−1.1 to 0.0)	0.054
Profession				
Senior officials and management	479	27.3 ± 8.61	−2.0 (−2.9 to −1.1)	<0.0001*
Professionals and technicians	2815	27.7 ± 9.41	−1.6 (−2.0 to −1.1)	<0.0001*
Clerical support workers	1291	28.4 ± 9.79	−0.9 (−1.5 to −0.3)	0.004*
Services and sales workers	4534	29.3 ± 9.85	Reference	Reference
Manual labour	1773	27.3 ± 9.50	−2.0 (−2.5 to −1.5)	<0.0001*
Elementary occupation	904	28.2 ± 9.95	−1.1 (−1.8 to −0.4)	0.003*
Disposable income				
<20th percentile	3183	28.7 ± 10.56	Reference	Reference
20–50th percentile	4442	28.6 ± 10.04	−0.1 (−0.6 to 0.4)	0.711
50–80th percentile	3982	28.1 ± 9.30	−0.6 (−1.1 to −0.2)	0.007
>80th percentile	1518	27.1 ± 9.07	−1.6 (−2.2 to −1.0)	<0.0001*
Residence				
Large city and municipality	4930	27.5 ± 9.66	Reference	Reference
Medium-sized town and municipality	4260	28.8 ± 10.08	1.2 (0.8–1.6)	<0.0001*
Small town, urban area, rural municipality	4070	28.7 ± 9.81	1.2 (0.8–1.6)	<0.0001*
Marital status				
Married/partner	6012	28.0 ± 9.52	Reference	Reference
Divorced/widow/widower	2085	27.6 ± 9.91	−0.4 (−0.9 to 0.1)	0.103
Single	5167	29.0 ± 10.18	1.0 (0.7–1.4)	<0.0001*
Financial aid				
None	10,196	28.6 ± 9.57	Reference	Reference
Retirement pension	236	24.3 ± 9.78	−4.3 (−5.6 to −3.1)	<0.0001*
Disability pension/early retirement	2145	27.0 ± 10.48	−1.7 (−2.1 to −1.2)	<0.0001*
Social benefits	698	28.9 ± 11.29	0.3 (−0.4 to 1.1)	0.405
Heritage				
Swedish born, Swedish descendant	10,665	28.7 ± 9.86	Reference	Reference
Swedish born, non-Swedish descendant	709	28.8 ± 9.62	0.1 (−0.7 to 0.8)	0.844
Born outside Sweden	1889	25.9 ± 9.62	−2.7 (−3.2 to −2.3)	<0.0001*

Total weight loss at 5 years after surgery, presented as mean ± standard deviation. Beta-values (95% Confidence Intervals) estimated with univariable linear regression.

*Significant *p* value (*p* < 0.05) after correction for multiple calculations.

An occupation other than service and sales work, clerical support work or management, receiving financial aid, being a 1st generation immigrant, and disposable incomes in the lowest 20th, and highest 80th percentiles, older age, male gender, higher BMI, and obesity-related comorbidity (other than dyspepsia/GERD) were associated with a lower %EBMIL. After correction for multiple calculations, disposable income and receiving social benefits no longer remained significant factors (Table 2).

**Table 2:** Excess-BMI loss 5 years after surgery.

	*N*	%EBMIL	*B* (95% CI)	Unadjusted *p*
Age				
<30	1877	75.6 ± 27.22	Reference	Reference
30–40	3474	74.0 ± 25.87	−1.6 (−3.1 to −0.1)	0.032
40–50	4303	71.3 ± 25.97	− 4.3 (−5.7 to −2.8)	<0.0001*
50–60	2798	68.8 ± 25.16	−6.9 (−8.4 to −5.3)	<0.0001*
>60	823	64.3 ± 25.91	−11.3 (−13.5 to −9.1)	<0.0001*
BMI				
<40	4617	80.8 ± 28.29	Reference	Reference
40–50	7521	68.1 ± 23.51	−12.7 (−13.6 to −11.7)	<0.0001*
50–60	1048	58.2 ± 20.09	−22.6 (−24.4 to −20.8)	<0.0001*
>60	89	53.0 ± 22.58	−27.8 (−33.7 to −21.9)	<0.0001*
Sex				
Female	10,308	73.9 ± 26.44	Reference	Reference
Male	2967	63.9 ± 23.35	−10.0 (−11.1 to −9.0)	<0.0001*
Comorbidity				
Sleep apnoea	1275	64.9 ± 24.89	−7.5 (−9.0 to −6.0)	<0.0001*
Hypertension	3574	67.2 ± 25.52	−6.0 (−7.0 to −5.0)	<0.0001*
Diabetes	2604	64.6 ± 25.69	−8.8 (−9.9 to −7.7)	<0.0001*
Dyslipidaemia	1412	66.7 ± 26.13	−5.6 (−7.0 to −4.1)	<0.0001*
Dyspepsia/GERD	1053	71.1 ± 26.39	−0.6 (−2.3 to 1.0)	0.443
Depression	1731	68.8 ± 29.80	−3.3 (−4.6 to −2.0)	<0.0001*
Cardiovascular comorbidity	705	66.9 ± 27.05	−5.1 (−7.0 to −3.1)	<0.0001*
Education				
Primary education < 9 years	2232	71.3 ± 27.16	−0.6 (−1.8 to 0.6)	0.327
Secondary education	8154	71.9 ± 25.93	Reference	Reference
Higher education < 3 years	1408	71.1 ± 25.73	−0.8 (−2.3 to 0.6)	0.261
Higher education > 3years	1425	71.7 ± 25.63	−0.2 (−1.7 to 1.2)	0.758
Profession				
Senior officials and management	479	72.0 ± 23.85	−2.4 (−4.9 to 0.0)	0.051
Professionals and technicians	2815	70.9 ± 25.24	−3.5 (−4.7 to −2.3)	<0.0001*
Clerical support workers	1291	72.8 ± 26.25	−1.6 (−3.2 to 0.0)	0.054
Services and sales workers	4534	74.4 ± 26.29	Reference	Reference
Manual labour	1773	67.4 ± 24.37	−7.0 (−8.4 to −5.6)	<0.0001*
Elementary occupation	904	71.3 ± 26.86	−3.1 (−5.0 to −1.2)	0.001*
Disposable income				
<20th percentile	3183	70.7 ± 27.38	Reference	Reference
20–50th percentile	4442	72.2 ± 26.50	1.5 (0.3–2.7)	0.015
50–80th percentile	3982	72.1 ± 25.19	1.4 (0.2–2.6)	0.027
>80th percentile	1518	71.0 ± 24.48	0.3 (−1.3 to 2.0)	0.417
Residence				
Large city and municipality	4930	71.4 ± 26.10	Reference	Reference
Medium-sized town and municipality	4260	71.6 ± 26.13	0.2 (−0.9 to 1.3)	0.706
Small town, urban area, rural municipality	4070	72.0 ± 26.12	0.7 (−0.4 to 1.8)	0.207
Marital status				
Married/partner	6012	72.1 ± 25.70	Reference	Reference
Divorced/widow/widower	2085	71.2 ± 26.65	−0.9 (−2.2 to 0.4)	0.181
Single	5167	71.3 ± 26.38	−0.8 (−1.8 to 0.2)	0.099
Financial aid				
None	10,196	72.5 ± 25.35	Reference	Reference
Retirement pension	236	62.4 ± 25.02	−10.2 (−13.4 to −6.9)	<0.0001*
Disability pension/early retirement	2145	69.1 ± 28.29	−3.5 (−4.7 to −2.2)	<0.0001*
Social benefits	698	69.8 ± 29.25	−2.7 (−4.7 to −0.7)	0.007
Heritage				
Swedish born, Swedish descendant	10,665	72.5 ± 26.16	Reference	Reference
Swedish born, non-Swedish descendant	709	72.2 ± 24.91	−0.3 (−2.3 to 1.7)	0.752
Born outside Sweden	1889	66.4 ± 25.69	−6.2 (−7.5 to 4.9)	<0.0001*

Excess-BMI loss at 5 years after surgery presented as mean ± standard deviation. Beta-values (95% Confidence Intervals) estimated with univariable linear regression.

*Significant *p* value (*p* < 0.05) after correction for multiple calculations.

After adjustment for factors previously known to affect weight loss after bariatric surgery (age, BMI, sex, and obesity-related comorbidities), higher education, living in larger cities and being a first-generation immigrant were independently associated with a lower %TWL and %EBMIL. An occupation as a professional or technician and receiving social benefits were independently associated with a lower %TWL, but not independently associated with a lower %EBMIL. After correction for multiple calculations, place of residence and being a first-generation immigrant remained significant risk factors (Table 3).

**Table 3:** Adjusted linear regression of total weight loss and excess BMI loss 5 years after surgery.

	*N*	%TWL		%EBMIL	
		*B* (95% CI)	Adjusted *p*^a^	B (95% CI)	Adjusted *p*^a^
Education					
Primary education < 9 years	2232	0.2 (−0.2–0.7)	0.296	0.8 (−0.3 to 2.0)	0.160
Secondary education	8154	Reference	Reference	Reference	Reference
Higher education < 3 years	1408	−0.6 (−1.1 to −0.1)	0.027	−1.6 (−2.9 to −0.2)	0.026
Higher education > 3years	1425	−0.7 (−1.2 to −0.1)	0.015	−1.5 (−2.9 to 0.2)	0.028
Profession					
Senior officials and management	479	0.0 (−0.9 to 1.0)	0.971	0.2 (−2.3 to 2.6)	0.893
Professionals and technicians	2815	−0.5 (−0.9 to 0.0)	0.039	−1.1 (−2.3 to 0.1)	0.074
Clerical support workers	1291	−0.2 (−0.8 to 0.4)	0.437	−0.3 (−1.8 to 1.2)	0.465
Services and sales workers	4534	Reference	Reference	Reference	Reference
Manual labour	1773	−0.1 (−0.8 to 0.5)	0.679	−0.5 (−2.2 to 1.2)	0.555
Elementary occupation	904	−0.2 (−0.9 to 0.5)	0.566	−0.2 (−2.0 to 1.5)	0.794
Disposable income					
<20th percentile	3183	Reference	Reference	Reference	Reference
20–50th percentile	4442	0.2 (−0.2 to 0.7)	0.333	0.4 (−0.8 to 1.5)	0.547
50–80th percentile	3982	0.0 (−0.4 to 0.5)	0.854	−0.1 (−1.3 to 1.1)	0.879
>80th percentile	1518	−0.1 (−0.8 to 0.5)	0.729	0.1 (−1.5 to 1.7)	0.896
Residence					
Large city and municipality	4930	Reference	Reference	Reference	Reference
Medium-sized town and municipality	4260	0.8 (0.4–1.2)	0.0001*	1.8 (0.8–2.8)	0.0006*
Small town, urban area, rural municipality	4070	0.8 (0.4–1.2)	<0.0001*	1.9 (0.9–2.9)	0.0002*
Marital status					
Married/partner	6012	Reference	Reference	Reference	Reference
Divorced/widow/widower	2085	0.1 (−0.4 to 0.5)	0.806	0.1 (−1.1 to 1.3)	0.126
Single	5167	−0.3 (−0.7 to 0.1)	0.152	−0.6 (−1.6 to 0.4)	0.210
Financial aid					
None	10,196	Reference	Reference	Reference	Reference
Retirement pension	236	−0.5 (−1.7 to 0.8)	0.482	−1.9 (−5.1–1.4)	0.258
Disability pension/early retirement	2145	−0.3 (−0.8 to 0.1)	0.177	−0.5 (−1.7 to 0.7)	0.451
Social benefits	698	−0.8 (−1.5 to −0.0)	0.041	−1.4 (−3.2 to 0.5)	0.139
Heritage					
Swedish born, Swedish descendant	10,665	Reference	Reference	Reference	Reference
Swedish born, non-Swedish descendant	709	−0.5 (−1.2 to 0.2)	0.157	−1.3 (−3.1 to 0.5)	0.165
Born outside Sweden	1889	−2.4 (−2.9 to −1.9)	<0.0001*	−6.2 (−7.4 to −5.0)	<0.0001*

Results of linear regression models on the total weight loss and excess weight loss, 5 years after primary gastric bypass.

^a^Adjusted for age, preoperative BMI, sex, sleep apnoea, hypertension, T2DM, dyslipidaemia, dyspepsia/GERD, depression, cardiovascular comorbidity.

*Significant *p* value (*p* < 0.05) after correction for multiple calculations.

Receiving disability pension/early retirement, social benefits, and being a first-generation immigrant, were all independently associated with a lower chance of achieving a postoperative EBMIL ≥ 50%, while employment as a senior official or manager, higher income, and residence in small towns were associated with a higher chance (Table 4).

**Table 4:** Proportion reaching excess BMI > 50% at 5 years after surgery.

	*N*	EBMIL > 50%	Unadjusted OR (95% CI)	Adjusted OR^a^ (95% CI)	adjusted *p*^a^
Age					
<30	1877	1553 (82.7%)	Reference		
30–40	3474	2862 (82.4%)	0.98 (0.84–1.13)		
40–50	4303	3402 (79.1%)	0.79 (0.68–0.91)		
50–60	2798	2167 (77.4%)	0.72 (0.62–0.83)		
>60	823	588 (71.4%)	0.52 (0.43–0.63)		
BMI					
<40	4617	3992 (86.5%)	Reference		
40–50	7521	5855 (77.8%)	0.55 (0.50–0.61)		
50–60	1048	676 (64.5%)	0.28 (0.24–0.33)		
>60	89	49 (55.1%)	0.19 (0.13–0.29)		
Sex					
Female	10,308	8430 (81.8%)	Reference		
Male	2967	2142 (72.2%)	0.58 (0.53–0.64)		
Comorbidity, *n* (%)					
Sleep apnoea, *n* (%)	1275	913 (71.6%)	0.61 (0.54–0.70)		
Hypertension, *n* (%)	3574	2647 (74.1%)	0.64 (0.58–0.70)		
Diabetes, *n* (%)	2604	1828 (70.2%)	0.52 (0.47–0.57)		
Dyslipidaemia, *n* (%)	1412	1021 (72.3%)	0.63 (0.56–0.72)		
Dyspepsia/GERD, *n* (%)	1053	820 (77.9%)	0.89 (0.77–1.04)		
Depression, *n* (%)	1731	1256 (72.6%)	0.63 (0.56–0.71)		
Cardiovascular comorbidity, *n* (%)	705	520 (73.8%)	0.70 (0.59–0.84)		
Education					
Primary education < 9 years	2232	1746 (78.2%)	0.89 (0.79–1.00)	0.98 (0.87–1.10)	0.733
Secondary education	8154	6535 (80.1%)	Reference	Reference	Reference
Higher education < 3 years	1408	1112 (79.0%)	0.93 (0.81–1.07)	0.89 (0.77–1.02)	0.096
Higher education > 3years	1425	1146 (80.4%)	1.02 (0.88–1.17)	0.93 (0.80–1.08)	0.321
Profession					
Senior officials and management	479	405 (84.6%)	1.19 (0.92–1.54)	1.42 (1.08–1.87)	0.011
Professionals and technicians	2815	2253 (80.0%)	0.87 (0.77–0.98)	1.01 (0.86–1.15)	0.864
Clerical support workers	1291	1031 (79.9%)	0.86 (0.74–1.00)	0.94 (0.80–1.11)	0.475
Services and sales workers	4534	3726 (82.2%)	Reference	Reference	Reference
Manual labour	1773	1350 (76.1%)	0.69 (0.61–0.79)	1.04 (0.89–1.22)	0.611
Elementary occupation	904	713 (78.9%)	0.81 (0.68–0.97)	0.97 (0.80–1.16)	0.712
Disposable income					
<20th percentile	3183	2464 (77.4%)	Reference	Reference	Reference
20–50th percentile	4442	3537 (79.6%)	1.14 (1.02–1.27)	1.06 (0.94–1.19)	0.359
50–80th percentile	3982	3221 (80.9%)	1.24 (1.10–1.39)	1.11 (0.99–1.26)	0.085
>80th percentile	1231	1231 (81.1%)	1.25 (1.07–1.46)	1.20 (1.02–1.41)	0.026
Residence					
Large city and municipality	4930	3920 (79.5%)	Reference	Reference	Reference
Medium-sized town and municipality	4260	3367 (79.0%)	0.97 (0.88–1.07)	1.07 (0.96–1.19)	0.230
Small town, urban area, rural municipality	4070	3270 (80.3%)	1.05 (0.95–1.17)	1.12 (1.01–1.25)	0.039
Marital status					
Married/partner	6012	4841 (80.5%)	Reference	Reference	Reference
Divorced/widow/widower	2085	1649 (79.1%)	0.91 (0.81–1.03)	0.99 (0.87–1.12)	0.821
Single	5167	4071 (78.8%)	0.90 (0.82–0.99)	0.91 (0.82–1.01)	0.067
Financial aid					
None	10,196	8292 (81.3%)	Reference	Reference	Reference
Retirement pension	236	166 (70.3%)	0.54 (0.41–0.72)	0.84 (0.63–1.14)	0.267
Disability pension/early retirement	2145	1591 (74.2%)	0.66 (0.59–0.74)	0.81 (0.71–0.91)	0.0004
Social benefits	698	523 (74.9%)	0.69 (0.57–0.82)	0.75 (0.62–0.91)	0.003
Heritage					
Swedish born, Swedish descendant	10,665	8604 (80.7%)	Reference	Reference	Reference
Swedish born, non-Swedish descendant	709	572 (80.7%)	1.00 (0.82–1.21)	0.94 (0.77–1.15)	0.561
Born outside Sweden	1889	1384 (73.3%)	0.66 (0.59–0.73)	0.64 (0.57–0.72)	<0.0001

Results of logistic regression model on the proportion of patients achieving satisfactory weight loss, defined as >50% excess BMI loss, 5 years after primary gastric bypass.

^a^Logistic regression model, adjusted for age, preoperative BMI, sex, sleep apnoea, hypertension, T2DM, dyslipidaemia, dyspepsia/GERD, depression, and cardiovascular comorbidity.

Amongst first-generation immigrants, all non-Nordic subgroups had less weight loss, in terms of both %TWL and %EBMIL. Patients born outside Europe also had a lower chance of achieving a postoperative EBMIL ≥ 50% (Table 5).

**Table 5:** Weight loss characteristics depending on place of origin.

Place of origin	*N*	%TWL	*p*^a^	%EBMIL	*p*^a^	EBMIL > 50%	*p*^a^
		Mean ± SD		Mean ± SD		*n* (%)	
Sweden	11,374	28.7 ± 9.84	Ref.	72.5 ± 26.09	Ref.	9176 (80.7%)	Ref.
Nordic countries	459	26.9 ± 9.72	0.249	67.5 ± 25.28	0.116	345 (75.2%)	0.211
Europe outside the Nordic countries	189	26.3 ± 9.79	0.006	66.9 ± 25.67	0.004	143 (75.7%)	0.111
Outside Europe	1241	25.5 ± 9.53	<0.001	65.8 ± 25.85	<0.001	896 (72.2%)	<0.001

Weight loss at 5 years after surgery, presented as total weight loss (%TWL), Excess-BMI loss (%EBMIL), and numbers reaching satisfactory weight loss (EBMIL > 50%). Specific country of birth was unknown for 12 patients.

^a^Adjusted linear regression (for %TWL and %EBMIL), and adjusted logistic regression (for EBMIL > 50%). All models adjusted for age, preoperative BMI, sex, sleep apnoea, hypertension, T2DM, dyslipidaemia, dyspepsia/GERD, depression, cardiovascular comorbidity.

No multicollinearity issue was detected in either of the multivariable models.

### Sensitivity analysis

Loss to follow-up was more common in patients with a low disposable income, those receiving social benefits, citizens of medium-sized towns, patients who were unmarried, patients with a higher BMI and younger ages, males, and those with absence of comorbidities (except for depression) (Supplementary Table 1). However, when entering only patients from centres with a >75% follow-up rate, very similar results to those of the main analyses were seen (Supplementary Table 2).

## Discussion

Among the socioeconomic variables studied, being a first-generation immigrant and living in a larger city were independently associated with less weight loss (measured by %TWL and %EBMIL).

With these exceptions, socioeconomic factors had less impact on weight loss than other patient-specific factors, which is consistent with previous smaller studies reporting a lack of association [11, 12].

First-generation immigrants experienced significantly less weight loss at 5 years than other groups of patients, and fewer patients in this group achieved satisfactory weight loss. After adjustment for other potential risk factors, the risk for less weight loss among patients born outside of the Nordic countries, and in particular outside of Europe, was equivalent to the effect of strong patient-demographic factors such as age, sex, and metabolic comorbidities. This group of patients may also experience higher complication rates [16] as well as less improvement in HRQoL [17]. Although there may be a difference in the response to bariatric surgery between ethnic groups [11, 24], the inferior weight loss among first-generation immigrants could be related to difficulties in their ability to understand and apply preoperative information (health literacy), failure to appreciate the importance of patient involvement, lack of a supportive network, and simple misunderstandings due to language or cultural mismatch between care providers and patients [25]. Furthermore, inherited eating habits and a different food culture could be of importance. Finally, the motivation of the patient to undergo bariatric surgery is known to differ [26, 27]. Although immigrants from countries outside of Europe had a tendency towards less weight loss, first-generation immigrants from other parts of Europe also achieved less weight loss than patients born in Sweden. This finding suggests a psychosocial rather than a strictly biological explanation for these differences in outcome.

Patients residing in larger cities had lost less weight 5 years after surgery than patients residing in small towns or municipalities. This group of patients has also been reported to be lost to follow-up more often and report less improvement in health-related quality of life after bariatric surgery [17, 28]. The explanation for this is likely to be multifactorial, including behavioural and sociopsychological factors not considered in the present study. Part of the explanation may lie in the chronic stress and higher cortisol levels associated with urban life [29], less time for exercise due to congestion, increased travelling times, as well as a higher availability of energy dense food, often called “junk food”.

In the unadjusted analyses, receiving social benefits were associated with less weight loss, and patients receiving social benefits or disability pension/early retirement were less likely to achieve satisfactory weight loss. Both groups are composed of individuals who often have a difficult economic situation and a higher proportion of physical or mental disabilities that influence their ability to follow diet and exercise recommendations postoperatively. Furthermore, these socioeconomically challenged patients often have a weaker social network and lower health literacy [30]. In fact, lower health literacy may contribute to poor outcome from non-communicable disease among socioeconomically weaker groups [31]. Moreover, a weak association was seen between education, profession and weight loss. Although this could be related to longer working hours and poor work–life balance, the slightly lower weight loss among patients with higher education and professionals/technicians contradicts previous reports and is likely to be due to inequality of access to bariatric surgery rather than a direct association [32].

In a previous American study on US veterans, the average income in the neighbourhood of the patient was reported to influence outcome after bariatric surgery [33]. In our study, higher personal income was associated with a slightly greater EBMIL but lower TWL, thus signalling a potential confounding effect of BMI. Indeed, after correction for other relevant factors, including BMI, no correlation was seen. The association between average neighbourhood income and bariatric surgery outcome is more likely to be explained by other factors associated with residence in poorer neighbourhoods, such as health literacy, lack of a supportive network, and poor access to healthcare. Indeed, it is known that patients with higher incomes have better access to bariatric surgery [34].

In addition to socioeconomic factors, several patient-specific factors also influenced 5-year weight loss. Older age, male gender, and obesity-related comorbidities other than dyspepsia/GERD were all associated with lower postoperative weight loss as well as a reduced chance of achieving satisfactory weight loss (EBMIL > 50%). Preoperative BMI had a strong impact on weight loss, but the impact of BMI was highly dependent on the outcome measured. When weight loss was measured as EBMIL, patients with a higher BMI at the time of surgery had less weight loss, which is in accordance with the results of several previous studies addressing EMBIL as an outcome measure [5, 7, 8, 35]. On the other hand, patients with a higher preoperative BMI lost a greater proportion of their total weight, supporting the results of studies using total weight loss as an outcome measure [6]. Given the link between TWL and other outcomes after bariatric surgery [36], both differences in total weight as well as excess BMI need to be considered when evaluating weight loss after bariatric surgery.

The greater weight loss among younger patients and those without obesity-related comorbidities is in-line with previous studies [5, 7, 9] and may be related to other factors, such as mobility, covariation with other risk factors (such as comorbid disease and age), and established insulin resistance with higher circulating insulin levels, as well as to the effects of medication on weight gain. Clinical depression has also been reported to be associated with poorer follow-up attendance, which in turn is known to be associated with poorer long-term weight results [28, 37].

Women had significantly greater weight loss and more often experienced satisfactory weight loss after surgery than men. Although this result contradicts the result of a recent Swiss study including 444 patients [6], it is supported by older studies [11]. Women also attend follow-up visits more often than men [28] and experience better improvement in health-related quality of life [17]. The better compliance and results among women may well be the result of different motivations for surgery. Furthermore, preoperative information, perioperative care, and long-term follow-up programmes are likely to be more adapted to suit the needs of women, since more women than men undergo bariatric surgery.

Although several groups with postoperative weight loss less than the mean were identified in this study, it is important to point out that all subgroups showed good weight loss results, confirming the benefits of bariatric surgery. The relatively poor weight loss results among certain subgroups warrant further research to gain more information about specific reasons. Meanwhile, since several of the groups experiencing a poorer weight-related outcome also tended to miss follow-up visits [28], bariatric surgical centres should concentrate on improving follow-up attendance rates, motivating and supporting these patients, and adapting follow-up programmes to meet the requirements of individual patients. The results of the present study suggest that certain socioeconomic groups, in particular first-generation immigrants, are at particular risk for poorer outcome and are a group likely to benefit from more intense perioperative support, as well as directed information adapted to cultural aspects and native language.

### Strengths and limitations

The major strengths of this study lie in the large number of patients included and the high quality of data. Furthermore, most previous studies have only measured weight loss as either TWL or EBMIL, but as evident in the present study, both measures are highly dependent on preoperative BMI, though in different ways. EBMIL allows comparisons of patients with varying initial and excess weights, but has the disadvantage of underestimating successful weight loss in patients with very high BMIs. TWL may be a better option under these circumstances, but it may not always provide sufficient clinically relevant information to reflect weight loss success or failure [38]. The inclusion of both measures in this study is thus a strength. There are, however, limitations that must be acknowledged. There were many patients whose weight at the 5-year follow-up was not registered. Maintaining a high follow-up rate over a long period after bariatric surgery is a great challenge [39]. For the purposes of research and patient well-being, however, follow-up is important since patients lost to follow-up are often those with inferior weight loss [28]. Even though a second analysis including only centres with high follow-up rates showed very similar results, the high loss to follow-up may still constitute a potential source of bias. The present study was also limited to socioeconomic and demographic definitions that were decided prior to starting the study. For this reason, cognitive and behavioural factors known to influence weight loss could not be evaluated [40, 41].

## Conclusion

All socioeconomic groups experienced improvements in weight after bariatric surgery. However, as first-generation immigrants and residents of larger cities tend to have inferior weight loss, increased support in the pre- and postoperative setting for these two groups could be of value. The remaining socioeconomic factors appear to have a weaker association with postoperative weight loss.

## Supplementary information

Supplementary Table 1

Supplementary Table 2
