# Supplementary material for: The association between socioeconomic factors and weight loss 5 years after gastric bypass surgery
Source: Int J Obes (Lond). 2020 Jul 10;44(11):2279–90. doi: 10.1038/s41366-020-0637-0 (PMC7577856; doi:10.1038/s41366-020-0637-0)
Supplement: Supplementary file 1 — Supplementary Table 1 [file 41366_2020_637_MOESM1_ESM.docx]

| **Supplementary Table 1. Risk for loss to follow-up at 5 years after surgery** | | | |
| --- | --- | --- | --- |
|  | Loss to follow-up  n (%) | OR (95%CI) | unadjusted-P |
| Age |  |  |  |
| <30 | 2317 (55.2%) | Reference | Reference |
| 30-40 | 3517 (50.3%) | 0.82 (0.76-0.89) | <0.0001 |
| 40-50 | 3652 (45.9%) | 0.69 (0.64-0.74) | <0.0001 |
| 50-60 | 1784 (38.9%) | 0.52 (0.47-0.56) | <0.0001 |
| >60 | 317 (27.8%) | 0.31 (0.27-0.36) | <0.0001 |
| BMI |  |  |  |
| <40 | 3902 (45.8%) | Reference | Reference |
| 40-50 | 6513 (46.4%) | 1.02 (0.97-1.08) | 0.377 |
| 50-60 | 1072 (50.6%) | 1.21 (1.10-1.33) | <0.0001 |
| >60 | 100 (52.9%) | 1.33 (1.00-1.77) | 0.053 |
| Sex |  |  |  |
| Female | 8638 (45.6%) | Reference | Reference |
| Male | 2949 (49.8%) | 1.19 (1.12-1.26) | <0.0001 |
| Comorbidity |  |  |  |
| Sleep apnoea | 929 (42.2%) | 0.82 (0.75-0.90) | <0.0001 |
| Hypertension | 2553 (41.7%) | 0.77 (0.72-0.81) | <0.0001 |
| Diabetes | 1953 (42.9%) | 0.83 (0.78-0.89) | <0.0001 |
| Dyslipidaemia | 940 (40.0%) | 0.74 (0.68-0.81) | <0.0001 |
| Dyspepsia/GERD | 773 (42.3%) | 0.83 (0.75-0.91) | 0.0001 |
| Depression | 1569 (47.5%) | 1.04 (0.97-1.12) | 0.245 |
| Cardiovascular comorbidity | 464 (39.7%) | 0.74 (0.66-0.84) | <0.0001 |
| Education |  |  |  |
| Primary education <9 years | 6879 (45.8%) | 1.12 (1.05-1.20) | 0.001 |
| Secondary education | 2109 (48.6%) | Reference | Reference |
| Higher education <3 years | 1235 (46.7%) | 1.04 (0.96-1.13) | 0.357 |
| Higher education >3years | 1293 (47.6%) | 1.08 (0.99-1.17) | 0.081 |
| Profession |  |  |  |
| Senior officials and management | 401 (45.6%) | 0.94 (0.82-1.09) | 0.419 |
| Professionals and technicians | 2286 (44.8%) | 0.92 (0.85-0.98) | 0.013 |
| Clerical support workers | 931 (41.9%) | 0.81 (0.74-0.89) | <0.0001 |
| Services and sales workers | 4020 (47.0%) | Reference | Reference |
| Manual labour | 1547 (46.6%) | 0.98 (0.91-1.07) | 0.696 |
| Elementary occupation | 761 (45.7%) | 0.95 (0.85-1.05) | 0.335 |
| Disposable income |  |  |  |
| <20th percentile | 3250 (50.5%) | Reference | Reference |
| 20-50 th percentile | 3692 (45.4%) | 0.81 (0.76-0.87) | <0.0001 |
| 50-80 th percentile | 3267 (45.1%) | 0.80 (0.75-0.86) | <0.0001 |
| >80th percentile | 1261 (45.4%) | 0.81 (0.74-0.89) | <0.0001 |
| Residence |  |  |  |
| Large city and municipality | 3850 (43.8%) | Reference | Reference |
| Medium-sized town and municipality | 4510 (51.4%) | 1.36 (1.28-1.44) | <0.0001 |
| Small town, urban area, rural municipality | 3189 (43.9%) | 1.00 (0.94-1.07) | 0.917 |
| Marital status |  |  |  |
| Married/partner | 4651 (43.6%) | Reference | Reference |
| Divorced/widow/widower | 1773 (46.0%) | 1.10 (1.02-1.18) | 0.012 |
| Single | 5133 (49.8%) | 1.28 (1.22-1.36) | <0.0001 |
| Financial aid |  |  |  |
| None | 9059 (47.0%) | Reference | Reference |
| Retirement pension | 84 (26.3%) | 0.40 (0.31-0.51) | <0.0001 |
| Disability pension/early retirement | 1480 (40.8%) | 0.78 (0.72-0.83) | <0.0001 |
| Social benefits | 964 (58.0%) | 1.55 (1.40-1.72) | <0.0001 |
| Heritage |  |  |  |
| Swedish born, Swedish descendant | 9316 (46.6%) | Reference | Reference |
| Swedish born, non-Swedish descendant | 634 (47.2%) | 1.02 (0.92-1.14) | 0.678 |
| Born outside Sweden | 1605 (45.9%) | 0.97 (0.91-1.05) | 0.452 |
