# Supplementary material for: The association between socioeconomic factors and weight loss 5 years after gastric bypass surgery
Source: Int J Obes (Lond). 2020 Jul 10;44(11):2279–90. doi: 10.1038/s41366-020-0637-0 (PMC7577856; doi:10.1038/s41366-020-0637-0)
Supplement: Supplementary file 2 — Supplementary Table 2 [file 41366_2020_637_MOESM2_ESM.docx]

| **Supplementary Table 2. Percentage total weight loss and Excess BMI-loss at 5 years after surgery at centers with follow-up rates ≥75%** | | | | | |
| --- | --- | --- | --- | --- | --- |
|  | N | %EBMIL | P | %TWL | P |
| Age |  |  |  |  |  |
| <30 | 594 | 74.4 ± 27.01 | Reference | 30.7 ± 10.36 | Reference |
| 30-40 | 1024 | 74.3 ± 25.39 | 0.421 | 30.3 ± 9.94 | 0.915 |
| 40-50 | 1214 | 72.4 ± 25.37 | <0.0001 | 28.5 ± 9.48 | 0.122 |
| 50-60 | 759 | 70.0 ± 24.22 | <0.0001 | 27.2 ± 9.18 | 0.002 |
| >60 | 226 | 62.9 ± 23.69 | <0.0001 | 25.1 ± 9.49 | <0.0001 |
| BMI |  |  |  |  |  |
| <40 | 1260 | 81.2 ± 27.72 | Reference | 26.9 ± 9.28 | Reference |
| 40-50 | 2185 | 69.1 ± 23.15 | <0.0001 | 29.5 ± 9.74 | <0.0001 |
| 50-60 | 344 | 59.8 ± 20.53 | <0.0001 | 31.4 ± 10.55 | <0.0001 |
| >60 | 28 | 58.4 ± 20.83 | <0.0001 | 35.6 ± 12.55 | <0.0001 |
| Sex |  |  |  |  |  |
| Female | 2939 | 74.5 ± 25.90 | Reference | 29.5 ± 9.84 | Reference |
| Male | 878 | 64.6 ± 22.37 | <0.0001 | 26.6 ± 9.40 | <0.0001 |
| Comorbidity |  |  |  |  |  |
| Sleep apnoea | 454 | 64.8 ± 24.22 | <0.0001 | 26.5 ± 9.61 | <0.0001 |
| Hypertension | 1001 | 67.8 ± 24.89 | <0.0001 | 26.8 ± 9.36 | <0.0001 |
| Diabetes | 781 | 65.8 ± 25.64 | <0.0001 | 25.6 ± 9.83 | <0.0001 |
| Dyslipidaemia | 354 | 65.3 ± 25.79 | <0.0001 | 25.6 ± 9.81 | <0.0001 |
| Dyspepsia/GERD | 303 | 71.4 ± 25.84 | 0.127 | 28.0 ± 9.99 | 0.552 |
| Depression | 446 | 67.3 ± 28.84 | <0.0001 | 26.9 ± 10.95 | <0.0001 |
| Cardiovascular comorbidity | 215 | 67.6 ± 25.97 | 0.010 | 27.2 ± 9.378 | 0.007 |
| Education |  |  |  |  |  |
| Primary education <9 years | 684 | 71.2 ± 26.34 | 0.326 | 29.0 ± 9.84 | 0.190 |
| Secondary education | 2366 | 72.6 ± 25.47 | Reference | 28.6 ± 10.07 | Reference |
| Higher education <3 years | 403 | 72.3 ± 24.18 | 0.367 | 28.6 ± 9.49 | 0.835 |
| Higher education >3years | 347 | 71.3 ± 25.07 | 0.204 | 28.3 ± 9.41 | 0.368 |
| Profession |  |  |  |  |  |
| Senior officials and management | 125 | 71.3 ± 20.67 | 0.071 | 27.9 ± 8.38 | 0.193 |
| Professionals and technicians | 731 | 71.8 ± 24.88 | 0.011 | 28.4 ± 9.43 | 0.026 |
| Clerical support workers | 351 | 73.0 ± 26.96 | 0.273 | 28.9 ± 10.17 | 0.375 |
| Services and sales workers | 1415 | 74.4 ± 25.82 | Reference | 29.5 ± 9.85 | Reference |
| Manual labour | 476 | 68.6 ± 23.40 | 0.007 | 28.1 ± 9.23 | <0.0001 |
| Elementary occupation | 270 | 73.2 ± 25.93 | 0.709 | 29.3 ± 9.89 | 0.486 |
| Disposable income |  |  |  |  |  |
| <20th percentile | 958 | 70.8 ± 26.59 | Reference | 29.2 ± 10.49 | Reference |
| 20-50 th percentile | 1334 | 72.7 ± 26.28 | 0.081 | 29.1 ± 10.08 | 0.902 |
| 50-80 th percentile | 1068 | 73.2 ± 24.07 | 0.031 | 28.7 ± 9.08 | 0.313 |
| >80th percentile | 421 | 71.5 ± 23.81 | 0.634 | 27.7 ± 9.03 | 0.017 |
| Residence |  |  |  |  |  |
| Large city and municipality | 1617 | 71.8 ± 25.10 | Reference | 28.2 ± 9.61 | Reference |
| Medium-sized town and municipality | 662 | 70.2 ± 26.00 | 0.162 | 29.3 ± 10.40 | 0.020 |
| Small town, urban area, rural municipality | 1532 | 73.4 ± 25.55 | 0.084 | 29.3 ± 9.73 | 0.001 |
| Marital status |  |  |  |  |  |
| Married/partner | 1694 | 72.3 ± 25.00 | Reference | 28.5 ± 9.57 | Reference |
| Divorced/widow/widower | 626 | 72.0 ± 25.83 | 0.769 | 28.2 ± 9.83 | 0.451 |
| Single | 1492 | 72.1 ± 25.85 | 0.848 | 29.5 ± 10.05 | 0.006 |
| Financial aid |  |  |  |  |  |
| None | 2905 | 73.1 ± 24.81 | Reference | 29.2 ± 9.51 | Reference |
| Retirement pension | 67 | 61.0 ± 24.44 | <0.0001 | 25.3 ± 10.60 | 0.001 |
| Disability pension/early retirement | 634 | 69.5 ± 27.07 | 0.001 | 27.4 ± 10.41 | <0.0001 |
| Social benefits | 211 | 71.6 ± 28.34 | 0.426 | 30.1 ± 11.03 | 0.174 |
| Heritage |  |  |  |  |  |
| Swedish born, Swedish descendant | 3004 | 72.8 ± 25.35 | Reference | 29.2 ± 9.78 | Reference |
| Swedish born, non-Swedish descendant | 217 | 75.2 ± 24.56 | 0.177 | 29.8 ± 9.06 | 0.414 |
| Born outside Sweden | 591 | 67.7 ± 25.91 | <0.0001 | 26.7 ± 9.96 | <0.0001 |
